# The spectrum of acute and chronic consequences of neurotrauma in professional and amateur boxing – A call to action is advocated to better understand and prevent this phenomenon

**DOI:** 10.1016/j.bas.2023.102743

**Published:** 2023-12-30

**Authors:** Michele Da Broi, Abdullah Al Awadhi, Philippe Voruz, Aria Nouri, Karl Schaller

**Affiliations:** a Division of Neurosurgery, Department of Clinical Neurosciences, Geneva University Hospitals, Geneva, Switzerland; b Clinical and Experimental Neuropsychology Laboratory, Faculty of Psychology, University of Geneva, Switzerland; c Department of Neuroscience, University of Geneva, Geneva, Switzerland

**Keywords:** Boxing, Traumatic brain injury, Cerebral hemorrhage, Acute subdural hematoma, Dementia pugilistica, Mortality

## Abstract

**Introduction:**

Despite changes in regulations, boxing-related injuries and fatalities are still occurring. The numbers available in the literature regarding mortality and long-term consequences may not accurately represent the actual situation. Indeed, the real extent of this phenomenon remains poorly known.

**Research question:**

Delineating the spectrum of acute and chronic consequences of boxing-related traumatic brain injuries (TBI).

**Material and methods:**

Narrative review of the literature concerning acute and chronic boxing-related TBI. Keywords such as mortality, boxing, subdural hematoma were used to search in PubMed and Google scholar. An updated analysis of the Velazquez fatalities collection in boxing was undertaken.

**Results:**

The Velazquez collection includes 2076 fatalities from 1720 to the present with a death rate of 10 athletes per year. More than half of the deaths (N = 1354, 65.2%) occurred after a knock-out, and nearly 75% happened during professional bouts. In Australia, from 1832 to 2020, 163 fatalities were recorded (75% professional). In Japan, from 1952 to 2016, 38 deaths were recorded with a mean age of 23.9 years. Up to 40% of retired professional boxers in the United States were diagnosed with symptoms of chronic brain injury. Clinical dementia is far more prevalent among professional boxers than in amateurs with an incidence of 20%.

**Discussion and conclusions:**

A concerted effort to raise awareness and shed light on boxing-related neuro-trauma is required. Similar considerations can be made for other combat sports or contact sports. A call to action to address this knowledge gap, decrease and prevent this phenomenon is advocated.

## Highlights

• Despite changes in regulations, boxing-related injuries and fatalities still occur.
• An unknown number of fatalities go undetected in unofficial bouts.
• Improved and systematic registration of boxing-related fatalities is mandatory.
• A call to action to decrease and prevent fatalities in boxing advocated.

## 1 Introduction

Boxing, like many other combat sports, requires that the winning party renders the opponent unable to defend themselves by inflicting the highest amount of head trauma as possible, optimally by knockout (KO). A KO occurs when a boxer falls to the ground and the referee counts to ten while the fighter is unable to get back on his feet. However, boxing-related neurotrauma presents as a vast spectrum, including acute, subacute, and chronic neurological and neuropsychological complications, as well as death. Despite changes in regulations, as well as improved medical and safety precautions, fatalities in the ring and long-term consequences of repeated traumatic brain injuries (TBI) are still occurring and reported in the literature (Donnelly et al., 2023; Alevras et al., 2022; Teramoto et al., 2018; Baird et al., 2010a). Over the years, the chronic effects of repeated TBI have become evident with the recognition of conditions such as chronic traumatic encephalopathy (CTE) or post-concussive syndrome (PCS), which dramatically impact quality of life (McKee et al., 2023; Iverson et al., 2023; Morales et al., 2022; Wallace et al., 2018). While CTE has received considerable media attention, the pathomechanism of it and underlying ring-related deaths following severe TBI remain poorly recognized. With regard to sudden ring side deaths, scientific evidence on this subject is practically non-existent. The most important source of epidemiological data on boxing-related acute deaths is the Velazquez fatality collection. It was created in the 50s based on clippings from boxing magazines, news articles or medical journal papers, and later updated and digitalized by Joseph Svinth (2023a, b). The collection reports 2076 fatalities deaths with a death rate of approximately 10 athletes per year. However, this number may not accurately reflect reality, since an unknown number of deaths go unnoticed, occurring in non-official bouts. Furthermore, while not “acute”, multiple significant TBIs can result in significant morbidity associated with neuropsychological symptoms of PCS, and accelerated mortality (Rawlings et al., 2020; Baugh et al., 2012; Levitch et al., 2018; Di Virgilio et al., 2019).

With the present review, we want to reignite the interest on boxing-related neurotrauma to establish a comprehensive platform to understand and manage this problem. We will present an updated analysis of the Velazquez collection, after providing a brief history of boxing and the evolution of protective equipment. Then, we will discuss the spectrum of neurological consequences of TBI in boxing from acute to chronic injury, including death. Finally, we will highlight areas in urgent need of investigation.

## 2 Material and methods

A Narrative review of the literature concerning the spectrum of acute and chronic consequences of boxing-related TBI was undertaken. An extensive search of keywords such as mortality, boxing, acute subdural hematoma, concussion, neurological injury, etc. in PubMed and Google scholar was performed. Reference searches of the key articles discussing these topics were also performed. Moreover, an updated analysis of the Velazquez fatalities collection in boxing was undertaken. Variables analyzed included age at time of death, fatalities by historical period, geographical location, type of bout, deaths by round, and the association between fatalities and KO or technical knockout (TKO). To have the final version of the collection we contacted the author, namely Joseph R. Svinth on the April 2, 2023. The graphs were created with Microsoft Excel based on the data from the last version of the Velazquez collection of fatalities in boxing.

## 3 Origins of Boxing and Evolution of Protective Equipment

The origin of boxing is unknown but the earliest evidence comes from Egypt and Sumer in the third millennium BC (Mohamed, 2020). Thereafter, boxing spread, becoming part of the Olympic games in 688 BC. The athletes boxed without any pause until one of the opponents was defenseless. The first trace of regulation was the introduction of gloves in Minoan Crete between 1400 and 1500 BC (Hauser et al., 2023), although in modern boxing it was the British boxer Jack Broughton who introduced in 1743 the padded boxing gloves (mufflers) and codified a set of rules, namely a break in the fighting after a knockdown and no blows below the belt (Gee, 2023). Later, the Queensberry Rules were published in 1867 and their use became widespread towards the end of the 19^th^ century (Marquess of Queensberry rules |, 2023). They stipulated the wearing of boxing gloves, a round of 3 min’ duration followed by 1 min of rest and counting to ten after a knockdown. Since 1946, protective measures in amateur boxing have become increasingly more uniform, including for example, the wearing of a head guard, more heavily cushioned gloves, shorter and fewer rounds, stopping the bout in accordance with the “outclassed rule” if the point difference becomes too large (>20), and the option for a boxer to interrupt the bout himself. Interestingly, the ringside doctor cannot intervene in a professional bout and only the referee is allowed to stop the match (Jako, 2002).

In 1979, the Toughman Contest was founded in Bay City, Michigan by the boxing promoter, Art Dore (French, 2022). The most common format prescribes the use of standard amateur boxing rules, namely 16 ounces for each glove, protective headgear, and bouts made of 3 rounds of boxing. Generally, the participants of a Toughman Contest tournament are local residents of a particular city with no previous professional and limited amateur experience. However, formats can vary from state to state and are configured to each state's rules and regulations (The Original Toughman Contest West).

A special mention must be made for female boxing. Indeed, in ancient times women did not compete in boxing, as well as in most of other sports. During the 1700s, women boxers were often a novelty competing in contests staged in London. The 1904 Olympics featured women's boxing only as a display event. It is only in the 70s that women started to train seriously for the ring and to fight, even though it was difficult for them to get bouts and gain acceptance by the boxing establishment. Later, in 1993 USA Boxing sanctioned women's amateur boxing, and the International Boxing Association (AIBA) followed in 1994. Women's boxing became an official Olympic sport at the London 2012 Games. In amateur boxing, women follow the rules of men's boxing with a few exceptions—the rounds are shorter, and women wear breast protectors, with groin protection being optional (Hauser and Sammons, 2023).

The event that brought attention on ring-related fatalities was the death of Duk Koo Kim, a lightweight boxer from Korea who lost consciousness several minutes after having lost a bout by technical knockout (TKO) in the 14^th^ round in November 1982. This resulted in the reduction of the number of rounds from 15 to 12 during bouts by the World Boxing Council in 1983, using as a justification that a study with their medical advisors found out that fighters are most severely injured during rounds 13 through 15 (Borges and ESPN.com., 2007).

Nevertheless, in 2013 the AIBA faced controversy over the changes in the regulations of amateur boxing disallowing male senior boxers (participants older than 18 years old) to wear headguards during their bouts (Murray et al., 2015). According to Dickinson et al. safety concerns pertained as much to facial cuts as TBI (Dickinson and Rempel, 2016). Indeed, significantly more cuts occur without headgear (Jako, 2002; Bianco et al., 2013) and there can be an augmented risk of TBI from head clashes. This risk was described in a case report of ASDH following competition (Falvey and McCrory, 2015).

## 4 Neurological injury mechanisms in boxing

TBIs are classified according to the severity into mild (GCS 13–15), moderate (GCS 9–12), and severe (GCS<9) (Undén et al., 2013). The severity of neurological injury by a boxer's punch is related to the way the blow is delivered and how mechanical forces are transferred to the opponent's head. The force transmitted by a blow is directly proportional to the mass of the glove and the velocity of the swing, while it is inversely proportional to the total mass opposing the punch (Jordan, 2009).

Generally, there are two means of delivering concussive blows. The first one involves the boxer delivering enough translational acceleration which translated the head center of gravity and forces might sometimes reach critical levels for concussions. The second means involves rotational acceleration, which occurs with impacts taking advantage of the offset from the head center of gravity. For instance, the hook, namely a blow to the temple, imparts a significant amount of rotational acceleration. According to biomechanical studies, boxer's punches transfer lower translational forces than in American football collision, but much higher rotational forces which reach more frequently levels consistent with concussions (Viano et al., 2005). Walilko and Viano (Viano et al., 2005; Walilko et al., 2005) compared TBI in American football and boxing and found some substantial differences. In fact, the professional football player's head undergoes much higher translational accelerations which are more likely to result in cerebral concussion and more likely to result in the player being removed from the match. Therefore, the exposure to repeated TBI is limited. In boxing, where rotational forces transmitted by head blows are prevalent, there is a completely different pattern of TBI. This has important clinical implications, for example, ASDH are mostly seen in boxing, while are extremely rare in American football. Studies in animals indicate that ASDH result from the effects of rotational forces stretching the bridging veins that run between the dura and brain cortex (Jordan, 1987; Unterharnscheidt, 1972). Another possible explanation involves punches directly to the jaw causing flexion of the head and neck and consequent stretching of the bridging veins or impacts to the forehead which cause extension of the head and neck and therefore compression of the bridging veins (Viano et al., 2005). Furthermore, boxers are infrequently knocked out and thus able to continue fighting. This exposes boxers to repeated TBI which may lead to damage to cerebral structures, such as substantia nigra, cerebellum, and brain hemispheres, which correlates with pathological findings of CTE (Rabadi and Jordan, 2001; Jordan, 2000).

Boxing-related neurological injuries can be divided into acute and chronic forms. Acute ones manifest as direct consequences of a single fight or even a single blow. These include concussions, mild, moderate, and severe TBIs, which may lead to death. Chronic injuries are typically caused by repeated TBI over a longer period. More persistent TBI and chronic consequences of TBI can manifest clinically as PCS, second impact syndrome (SIS), and CTE.

### 4.1 Concussions, mild TBI (GCS 13–15) and neurological vulnerability

Concussions are the most common form of mild TBI in contact sports (Koh and Cassidy, 2004). According to a recent systematic review published by Donnelly et al. boxers have a significantly elevated risk of sustaining a concussion compared with other combat sports (Donnelly et al., 2023). What distinguishes concussions from mild TBI is that the first are defined as a functional injury without structural damages seen in conventional neuroimaging, whereas the latter may present with both structural and functional injury (Karlin, 2011). According to the 6^th^ international conference on concussion in sport, a sport-related concussion is defined as a traumatic brain injury caused by a direct blow to the head, neck or body resulting in an impulsive force being transmitted to the brain that occurs in sports and exercise-related activities (Patricios et al., 2023).

A recent paper by Hanell and Rostami describes three hypotheses (Hånell and Rostami, 2020), namely the convulsive, the vascular, and the mechanoporation hypothesis. Though none of the hypotheses can be considered fully verified, mechanoporation is the most consistent.

Pathophysiologically, biomechanical injury results in microstructural changes including indiscriminate glutamate release and perturbation of ionic flux, such as potassium efflux, and sodium and calcium influx. Homeostatic cell efforts result in hyperglycolysis with consequent relative depletion of intracellular energy reserves and accumulation of ADP. In the hyperacute phase, this increased demand for energy occurs in a setting of reduced cerebral blood flow producing a mismatch between energy demand and supply. Moreover, intracellular increased calcium concentration is accommodated by the sequestration of calcium into the mitochondria which may lead to mitochondrial dysfunction and exacerbation of the cellular energetic crisis. The initial hyperglycolitic period is followed by an impaired glucose metabolism which can last up to 7–10 days. The cellular energetic crisis, as well as the subsequent metabolic changes are thought to be underlying mechanisms of post-concussion vulnerability, leading to a less competent response to further injuries (Hånell and Rostami, 2020; Giza and Hovda, 2014).

Symptoms and signs may present immediately, or evolve over minutes or hours, and commonly resolve within days, but may be prolonged. The range of symptoms and signs is vast and that may or may not involve loss of consciousness. Furthermore, the clinical presentation of concussion cannot be explained solely by drug, alcohol, or medication use, other injuries (such as cervical injuries, peripheral vestibular dysfunction) or other comorbidities (such as psychological factors or coexisting medical conditions) (Patricios et al., 2023).

The recognition, evaluation, diagnosis, and management of sport-related concussion is complex. A concussion should be suspected every time one of the aforementioned clinical domains is affected; the athlete should then be removed from play and undergo prompt sideline evaluation using the Sport Concussion Assessment Tool 6 (SCAT6) or any other equivalent tool (Hall et al., 2005; Echemendia et al., 2023a, 2023b; Scorza and Cole, 2019).

### 4.2 Moderate (GCS 9–12), severe TBI (GCS <9) and death

In boxing, ASDH are the most common form of intracranial hemorrhage, as well as the most common life-threatening injury (Bailes and Cantu, 2001). ASDH seen in boxers are dissimilar from those diagnosed in the average population, since athletes generally have a smaller subdural space which leads to a more rapid increase in intracranial pressure. Whereas the incidence of subclinical intracranial hemorrhage in boxers is unknown, acute SDHs and associated cerebral edema are the leading cause of boxing-related death. Clinical consequences can be abrupt with loss of consciousness, development of focal neurological deficits, or in the worst case, death (Miele et al., 2006).

**Fig. 1:**
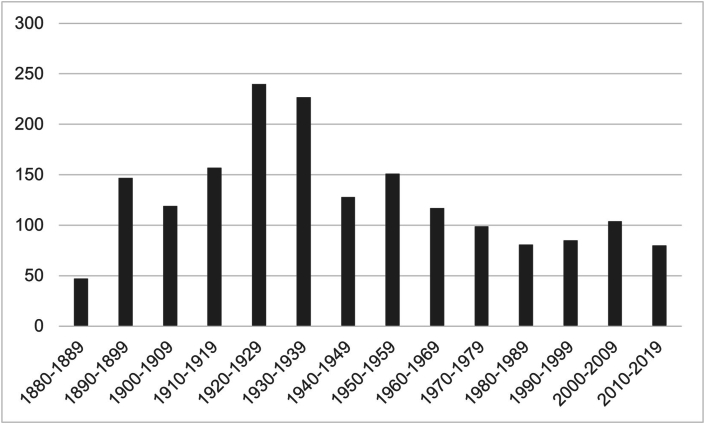
Bar graph showing fatalities by decades since 1880, namely after the bare-knuckle era of boxing and when most of fatalities were recorded.

Regarding ring-related deaths, the last version of the Velazquez collection includes 2076 fatalities. Two-hundred ninety-four of them (14.2%) happened between 1720 and 1880, namely during the bare-knuckle era of boxing, while most of them (N = 993, 47.8%) took place between 1880 and World War II. Before 1880, all the collection's deaths happened in English-speaking countries, mainly in England (N = 233/294, 79.3%), while thereafter, deaths started to be noted also in non-English-speaking countries like France, Italy, or Germany. Indeed, after World War II and the globalization of boxing, 789 deaths (38.0%) were registered, with 396 (50.2%) of them in non-English-speaking countries (Fig. 1).

The mean age of boxers at the time of death was 23 years of age (median age of 22 years). The youngest boxer died at 8 years of age during a sparring session in 1907 in Las Vegas, while the oldest was 91 years old (his opponent was 85 years old). According to the London Times newspaper article reported in the Velazquez collection, the two men decided to settle a dispute with a prizefight in 1822 in Gosford, England. The 91-year-old boxer won the bout, but he died of injuries a week later.

**Fig. 2:**
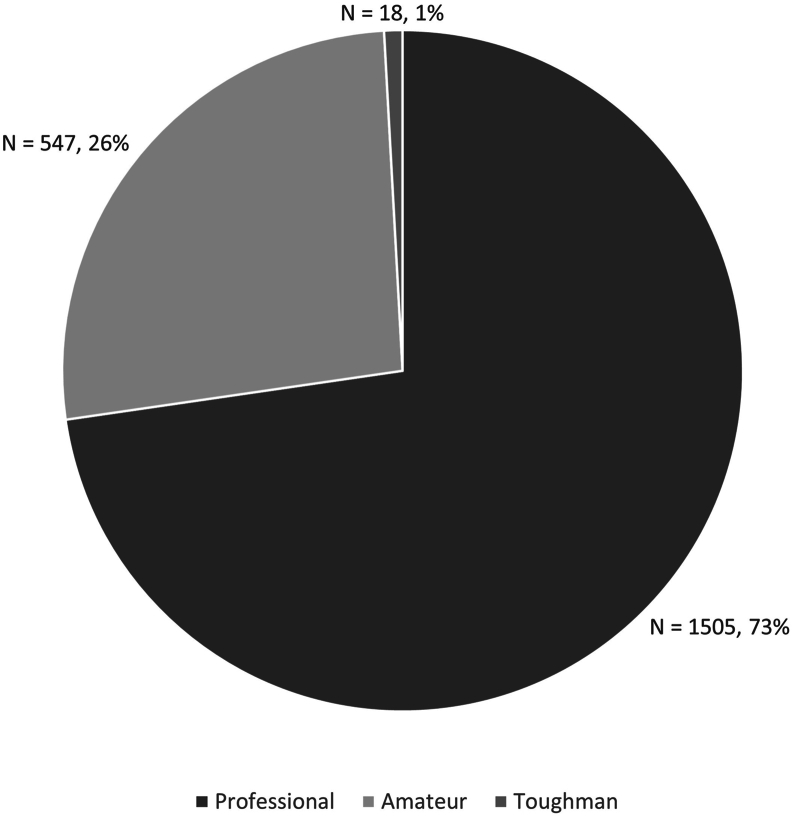
Pie chart showing the distribution of fatalities by bout type, namely professional, toughman, or amateur bout.

Nearly three quarters of fatalities (N = 1505, 73%) happened during professional bouts, while 26% (N = 547) of bouts were amateur, and only 1% (N = 18) were toughman bouts (Fig. 2).

**Fig. 3:**
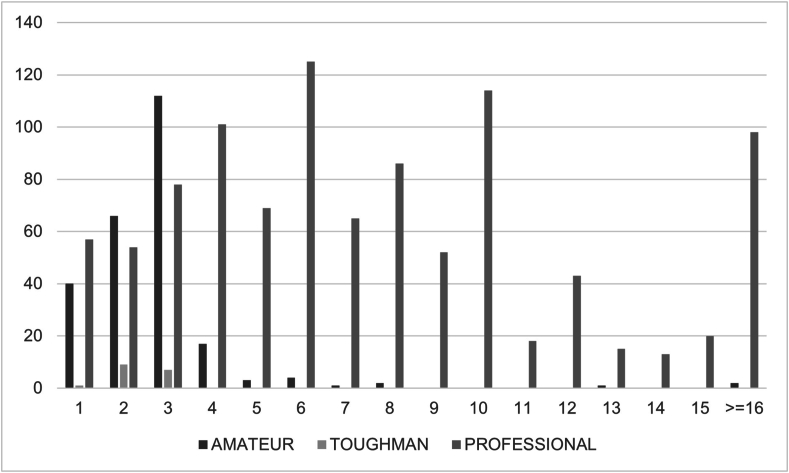
Bar graph showing fatalities by category, namely professional, toughman, and amateur and by round.

The highest mortality among professional bouts (N = 125/1008, 12.4%) was recorded at the 6^th^ round, followed by the 10^th^ round with 114 deaths (11.3%), and the 5^th^ round with 101 deaths (10.0%). The total number of deaths after the 12^th^ round is 189, accounting for 18.8% of all deaths registered for professional boxing. Considering the other categories, all deaths in the toughman bouts happened between the 1^st^ and the 3^rd^ round and concerning the amateur matches, the highest mortality was registered at the 1^st^, 2^nd^, and 3^rd^ round with 112/248 (45.2%), 66/248 (26.6%), and 40/248 (16.1%) deaths, respectively. Noteworthy, amateur boxing bouts consist of 3 3-min rounds (Hauser et al., 2023) (Fig. 3).

In 1134 cases (54.6%), the cause of death was due to a brain injury, but only in 267 cases (N = 267/1134, 23.5%) were recognized as due to a blow. The other causes of death were cardio-pulmonary (N = 191, 9.2%), cervical fractures (N = 27, 1.3%), or other causes (N = 724, 34.9%).

Concerning the location of preterminal event, most of the fatalities happened directly on the ring (N = 1670, 80.4%), in 313 cases (15.1%) death happened within the 24 h following the bout, and only in 89 cases (4.3%) the athlete died after 24 h from the bout. The location of death was not reported in 4 cases (0.2%).

Altogether, more than half of the fatalities (N = 1354, 65.2%) reported in the collection occurred after a KO and more than 10% (N = 233, 11.2%) occurred following a TKO. Specifically, after 1983, 137 deaths (6.6%) occurred after a KO, 65 (3.1%) after a TKO, 2 (0.1%) after a win TKO, 17 (0.8%) after a win decision, 26 (1.3%) after a loss decision, one after a “no decision”, and 6 (0.3%) were draws.

A decline in ring-related deaths was registered after 1983, when the number of rounds per bout in professional boxing was reduced from 15 to 12. However, no evidence was found to support the relationship between the reduction of number of rounds and the fall of boxing-related fatalities. Allegedly, this trend might be related to shorter careers and fewer fights, hence, reduced exposure to repeated TBIs (Baird et al., 2010b).

There are few studies in literature addressing mortality in boxing. According to Alevras et al. during 1832–2020 in Australia 163 boxing-related fatalities were recorded, namely 122 professional athletes and 40 amateur boxers. The most common cause of death was a TBI (74.2%). A decline in mortality was recorded from 1930 to 1980. Since the introduction of legislation to regulate boxing in the mid-70s, there were a total of 11 deaths, of which all but one were caused by TBI (Alevras et al., 2022). Teramoto et al. investigated boxing-related deaths according to changes in regulation that occurred in the mid-90s. Data were collected from 1952 to 2016 using the Velazquez collection, BoxRec ring fatalities and the yearly book published by the Japanese Boxing Commission. A total of 38 deaths was recorded with a mean age of 23.9 ± 3.3 years. Approximately 60% of the deceased boxers lost the final match, and 52.6% lost it by KO. According to their results, changing weigh-in to the day before the match and prohibiting 6-oz gloves did not result in reducing boxing fatalities in Japan (Teramoto et al., 2018).

### 4.3 Chronic TBI and CTE

Among chronic sequelae of repeated TBI, there are PCS, SIS, and CTE. PCS is characterized by the persistence of neurological symptoms of concussions lasting for more than 3 months (Hall et al., 2005). PCS is seen in 40–80% athletes exposed to repeated mild TBI and about 10–15% still report persistent neuropsychological symptoms after 1 year (Williams et al., 2010; Røe et al., 2009).

SIS has been defined as the following: “an athlete who has sustained an initial head injury, most often a concussion, sustains a second head injury before the symptoms associated with the first have fully cleared” (Cantu, 1996). Pathophysiologically, SIS is described as the rapid development of cerebral edema after a second TBI, usually a minor one (Giza and Hovda, 2001; Maroon et al., 2000). In severe cases, this may lead to malignant cerebral edema followed by brain herniation (Bey and Ostick, 2009; Byard and Vink, 2009). Along with the PCS, SIS is also related to the aforementioned post-concussion vulnerability (DeSalles et al., 1987). SIS was reported in 71% of American football players, 14% in boxing, and a few cases were reported in karate, skiing and ice hockey (Mori et al., 2006).

The most feared long-term consequence of repeated TBI is CTE, a neurodegenerative disorder which occurs several years after the career end. Risk factors for CTE include retirement after the age of 28 years, boxing career longer than 10 years, and participation in 150 or more bouts (Roberts, 1969). Agarwal et al. found that up to 40% of retired professional boxers in the United States were diagnosed with symptoms of chronic brain injury (Agarwal et al., 2023). A recently published case series including 152 brains of contact sport athletes younger than 30 years at the time of death highlighted several neuropathologic abnormalities associated with CTE. Particularly, these pathological anomalies included ventricular enlargement, cavum septum pellucidum, thalamic notching, and perivascular pigment-laden macrophage deposition in the frontal white matter. These findings confirm that CTE can be found also in young athletes. However, the clinical correlates are still uncertain (McKee et al., 2023).

From a neuropsychological perspective, memory and executive-attention deficits are expected (Heilbronner et al., 2009). Nevertheless, other functions could be impaired, comparable to the neuropsychological deficits observed in Alzheimer's disease (LoBue et al., 2020). According to the literature, clinical dementia is far more prevalent among professional boxers than in the amateur category and its incidence is approximately 20% (Jordan, 2000; Loosemore et al., 2007). Moreover, Orrison et al. found out that 76% of professional boxers showed abnormalities on cerebral MRI, such as hippocampal and cortical atrophy, dilated perivascular spaces, and diffuse axonal injury; these abnormalities also correlate with career length and the number of bouts (Orrison et al., 2009).

## 5 Perspectives and the need for further investigations

Since the dawn of the sport, when athletes boxed to exhaustion without any break and any protective gear, efforts to make boxing safer have been made. Nonetheless, the primary objective of the competition is inflicting trauma on the other individual. Therefore, while attempts should be made to reduce unnecessary injury to these athletes, certain levels of TBI must be accepted as this is inherent to the sport. It should be noted that, although KO are relatively uncommon, occurring in <5% of professional bouts, 64% of boxing-related fatalities registered since 1729 occurred after a KO showing a possible relationship between mortality and KO or TKO (Baird et al., 2010b). Further investigations are needed, such as athletes’ movement analyses and more biomechanical studies examining the impact of protective devices on reducing forces transmitted to the brain following a TBI.

Another important aspect are the long-term consequences of repeated TBI. These are less sensationalist and do not frequently make headlines (with some notable exceptions), but conditions such as CTE and PCS are a major health concern, and certainly result in a reduced quality of life and life expectancy. However, like acute death from boxing, mechanisms behind CTE remain incompletely understood, ultimately hindering efforts at prevention or treatment. Therefore, once CTE is suspected, early diagnosis utilizing advanced neuroimaging and transcranial non-invasive stimulation coupled with neurocognitive tests is essential (Heilbronner et al., 2009).

Given the much shorter history of female boxing, practically no data are available in the literature, except for some case reports (Miele et al., 2004). We believe that this represents an aspect that needs to be addressed in further studies and records should likewise and perhaps more urgently be undertaken for female boxers, given that TBI in females is becoming an increasingly recognized element in other contact sports. For instance, more data are available in the literature concerning soccer. In fact, according to a recent systematic review regarding sex-based differences for concussion incidence in soccer, the incidence rates are significantly higher in female soccer players compared to the male counterpart while heading (Dave et al., 2022).

In conclusion, a concerted effort to raise awareness and shed light on boxing-related neuro-trauma is clearly required, and as neurosurgeons, it is our role to take on this challenge. Similar considerations can be extended to other combat sports or contact sports, such as American football, where repeated TBI are also frequent. A call to action to address this knowledge gap, decrease and prevent this phenomenon is advocated.

## 6 Limitations

The Velazquez fatalities collection is the most important source of epidemiological data concerning acute deaths in boxing. However, due to the nature of the topic, the collection was not created by following scientific methodology. Indeed, information derives from newspaper articles and not from national databases or other verified sources under the control of an authority. Therefore, data may be incomplete or incorrect. A full analysis of the collection was not performed, since it was not the goal of this narrative review. Moreover, a more thorough analysis was already done by Baird and colleagues who are cited repeatedly in this paper. Our objective was to present some updated data on mortality in boxing. Furthermore, there was no female representation within this review because there was very little data available concerning acute or chronic consequences of boxing-related TBI in female amateur or professional boxers. This represents a limitation of our review study to highlight any gender discrepancies, including whether there is a gender-related neurological vulnerability.

## Ethics approval and consent to participate

Not applicable.

## Consent for publication

Not applicable.

## Availability of data and materials

Data sharing is not applicable to this article as no datasets were generated or analyzed during the current study.

## Funding

The authors declare that they did not receive any funding for this article.

## Author's contributions

Data collection: not applicable. Manuscript drafting: MDB. Critical revision: all authors. Manuscript approval: all authors. All authors have read and agreed to the published version of the manuscript.

## Declaration of interests

The authors declare that they have no known competing financial interests or personal relationships that could have appeared to influence the work reported in this paper.

## List of abbreviations

ASDH: Acute Subdural Hematoma
CTE: Chronic Traumatic Encephalopathy
GCS: Glasgow Coma Scale
KO: Knock-Out
PCS: Post-Concussive Syndrome
SCAT: Sport Concussion Assessment Tool
SIS: Second Impact Syndrome
TBI: Traumatic Brain Injury
TKO: Technical Knock-Out
